# Lymphatic platelet thrombosis limits bone repair by precluding lymphatic transporting DAMPs

**DOI:** 10.21203/rs.3.rs-3474507/v1

**Published:** 2023-11-14

**Authors:** Yong-Jun Wang, Yangkang Zheng, Lin Cong, Pengyu Wang, Li Zhao, Lianping Xing, Junling Liu, Hao Xu, Ning Li, Yongjian Zhao, Qi Shi, Qianqian Liang

**Affiliations:** Longhua Hospital, Shanghai University of Traditional Chinese Medicine, Shanghai, China; Longhua Hospital, Shanghai University of Traditional Chinese Medicine, Shanghai, China; Longhua Hospital, Shanghai University of Traditional Chinese Medicine, Shanghai, China; Longhua Hospital, Shanghai University of Traditional Chinese Medicine, Shanghai, China; Longhua Hospital, Shanghai University of Traditional Chinese Medicine, Shanghai, China; University of Rochester Medical Center; Shanghai Jiao Tong University School of Medcine; Longhua Hospital, Shanghai University of Traditional Chinese Medicine; Longhua Hospital, Shanghai University of Traditional Chinese Medicine; Longhua Hospital Shanghai University of Traditional Chinese Medicine; Longhua Hospital, Shanghai University of Traditional Chinese Medicine, Shanghai, China; Longhua Hospital, Shanghai University of Traditional Chinese Medicine

**Keywords:** Lymphatic platelet thrombosis, lymphatic drainage, fracture healing, immunomodulation, DAMPs

## Abstract

Lymphatic vessels (LVs) interdigitated with blood vessels, travel and form an extensive transport network in the musculoskeletal system. Blood vessels in bone regulate osteogenesis and hematopoiesis, however, whether LVs in bone affect fracture healing is unclear. Here, by near infrared indocyanine green lymphatic imaging (NIR-ICG), we examined lymphatic draining function at the tibial fracture sites and found lymphatic drainage insufficiency (LDI) occurred as early as two weeks after fracture. Sufficient lymphatic drainage facilitates fracture healing. In addition, we identified that lymphatic platelet thrombosis (LPT) blocks the draining lymphoid sinus and LVs, caused LDI and then inhibited fracture healing, which can be rescued by a pharmacological approach. Moreover, unblocked lymphatic drainage decreased neutrophils and increased M2-like macrophages of hematoma niche to support osteoblast (OB) survival and bone marrow-derived mesenchymal stem cell (BMSC) proliferation via transporting damage-associated molecular patterns (DAMPs). These findings demonstrate that LPT limits bone regeneration by blocking lymphatic drainage from transporting DAMPs. Together, these findings represent a novel way forward in the treatment of bone repair.

## INTRODUCTION

Even with improved methods of internal fixation and tissue engineering strategies, 5–10% of fractures still fail to heal properly while developing either nonunion or delayed union, resulting in an enormous financial burden on the healthcare system^1,2^. Therefore, the identification of a novel and optimal treatment for fractures remains an important unmet clinical need in orthopedics.

Blood vessels in bone regulate osteogenesis and hematopoiesis are widely reported^3–7^. Lymphatic vessels (LVs) interdigitated with blood vessels, travel and form an extensive transport network in the musculoskeletal system, especially that in the skin, muscle and periosteum^8–10^. Recently, Biswas et al. utilized light-sheet microscopy technology and identified lymphatic vessel network within bone marrow cavity and bones. They also demonstrate that radiation and chemotherapy induced injury model promote proliferating lymphatic endothelial cells to secrete CXCL12, which triggers expansion of mature Myh11 + CXCR4 + pericytes for bone and hematopoietic regeneration^10^. This brilliant and novel work reveals lymphangiocrine function relieved chemoradiotherapy induced bone injury. However, lymphatic drainage is one of essential biological functions in lymphatic system. Whether lymphatic draining function contributes bone fracture remains unknown.

Here, we monitored lymphatic draining function at the fracture sites in C57BL/6 mice with tibial fractures and revealed that lymphatic drainage insufficiency (LDI) occurred as early as two weeks after fracture. Stimulation of lymphatic drainage function by treatment with recombinant human VEGF-C protein improved fracture healing. We further investigated the cellular mechanisms leading to LDI during the early phase of fracture healing and identified that platelet thrombosis blocked the draining lymphoid sinus and LVs at upstream lymph node. Use of a low dose of Clopidogrel, a widely used blood thinner in clinical practice, reduced lymphatic platelet thrombosis (LPT) and improved fracture healing via increasing lymphatic drainage. At last, we elucidated the mechanism of lymphatic drainage regulated-bone repair that unblocked LVs immunomodulate hematoma niche to support osteoblast (OB) and bone marrow mesenchymal stem cells (BMSCs) via transporting damage associated molecular patterns (DAMPs).

## Results

### Impaired lymphatic draining function and enlarged draining lymph nodes in the fractured hindlimb

**Figure 1:**
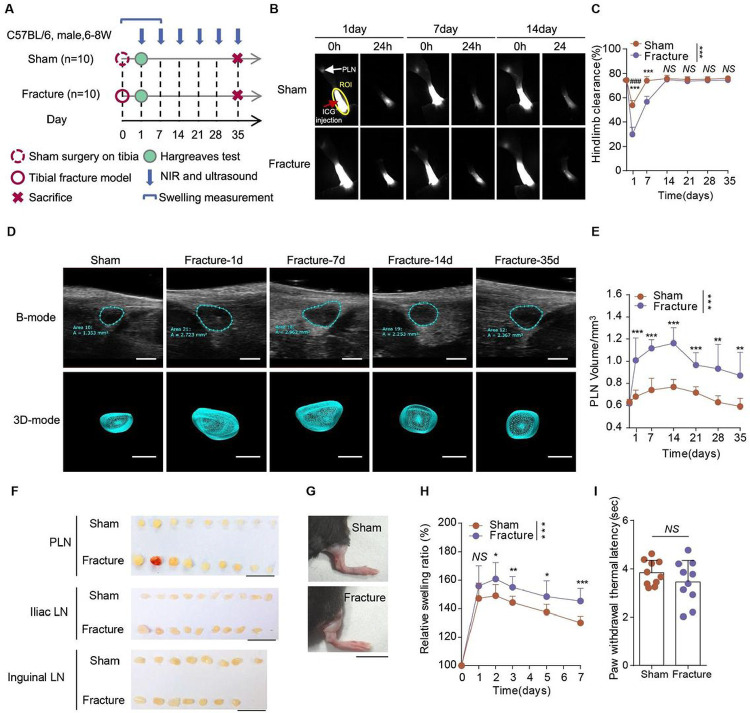
Impaired lymphatic draining function and enlarged draining lymph nodes in the fractured hindlimb. (**A**) Scheme of the experimental procedure for the fracture and sham groups. 6–8 weeks old male C57BL/6 mice received fracture or sham surgery of the right tibia and were sacrificed at day 35. (**B-C**) Draining collective LVs of the entire hindlimb examined by NIR-ICG imaging at 0 hour and 24 hours following ICG injection at different time points post fracture. (**B**) Representative NIR-ICG images at day 1, 7 and 14 post fracture surgery (n=10/group at each time point). The ICG fluorescence signal intensity at the footpad (outlined by yellow circle) was recorded. The red arrow indicates ICG injection site. The white arrow indicates the PLN. ROI = region of interest. (**C**) Quantitative analysis of lymphatic clearance in (**B**). (**D**) Temporalultrasound image of PLNs after fracture (n=10/group at each time point). Scale bars, 1 mm. (**E**) Quantitative analysis of the PLN volume in (**D**). (**F**) Gross anatomy of isolated PLNs, iliac LNs and inguinal LNs at day 35 post-surgery. Scale bars, 1 cm. (**G**) Representative photographs of the mouse hindlimb at day 2 post-surgery (n=10/group). Scale bar, 1 cm. (**H**) The relative swelling ratio of mouse hindlimb measured in 7 days post-surgery. (**I**) The paw withdrawal thermal latency (PWTL) at day 1 post-surgery. Data are means ± SD. *P < 0.05; **P<0.01; ***P < 0.001; NS, not significant. ###*P* < 0.001 vs day 1 by Paired t-test. n=10 per group. In **C**, **E** and **H**, analysis of variance in repeated measurement design followed the simple effect test. In **I**, two-sided Student’s t-test.

LDI with dilatation of the draining collecting lymphatics occurs after fractures^11,12^, but little is known about its onset and duration. To investigate the pathophysiological changes in lymphatic dysfunction at different phases of fracture healing, we performed open tibial fracture surgery or sham surgery on 6–8 week-old C57/BL6 male mice. We monitored lymphatic draining functions, including lymphatic clearance by NIR-ICG and volume of the PLN by ultrasound. We started these measurement on day 1 after surgery, and once a week for 35 days, a time covering the full course of fracture healing (Fig. 1A). We also measured post-fracture edema from day 0 to day 7 post-fracture and thermal pain in the fractured hindlimb using Hargreaves test at day 1 post-surgery for evaluating pain sensitivity.

NIR-ICG imaging showed that lymphatic clearance was reduced dramatically on day 1 post-surgery in both fracture and sham groups, though the extent of reduction was more in the fracture group than in the sham group (Fig. 1B–C). The lymphatic clearance of the fracture group remained significantly lower than the sham group (non-fracture) up to 1-week post-surgery, a time point when lymphatic clearance in the sham group had returned to non-fracture level (Fig. 1B–C). Ultrasound imaging revealed that the PLN volumes of the fracture group were significantly larger than that of the sham group during the whole process of fracture healing (Fig. 1D–E). At day 35 post-fracture, PLNs and iliac LNs isolated from the fracture group were larger than that from the sham group, while inguinal LNs did not show obvious differences (Fig. 1F). Of note, our previous study showed that ILNs drain the upper hindlimb area from the knee^13^. In addition, at day 7 post-surgery, the hindlimbs of the fracture group were more swollen than those of the sham group (Fig. 1G–H). Pain withdrawal thermal latency (PWTL) showed no significant difference between the two groups at day 1 post-surgery (Fig. 1L) as measured by a Hargreaves test. We consider both groups of mice suffer tissue injury and post-surgery induced pain though sham group didn`t experience bone fracture. These results suggest that fracturing of the hindlimb results in LDI and enlarged LNs shortly after injury, and LDI continues as long as 2 weeks post-fracture.

### Sufficient lymphatic drainage improves fracture healing

**Figure 2:**
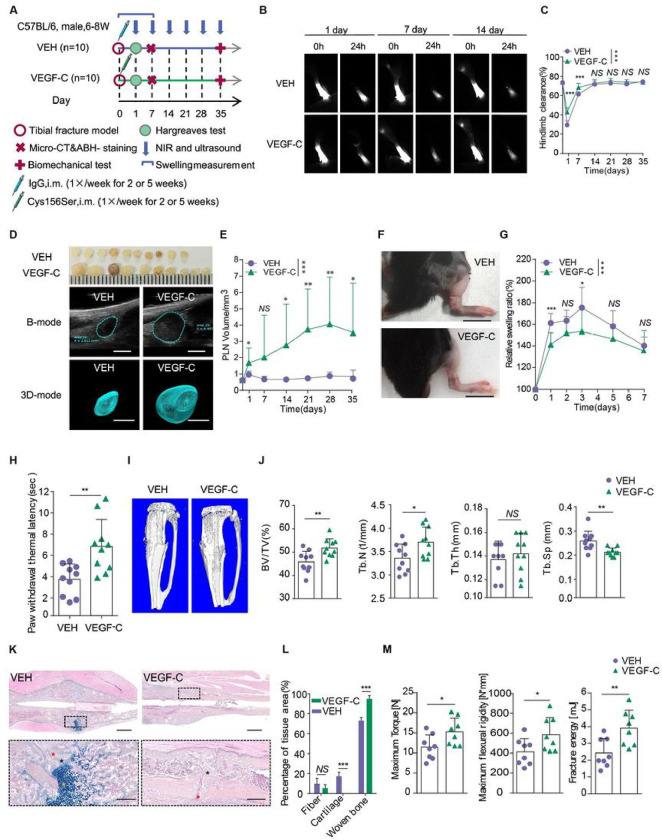
Sufficient lymphatic drainage improves fracture healing (**A**) Scheme of experimental procedure for VEGF-C therapy. After fracture, mice were intramuscularly injected with rh VEGF-C protein, Cys156Ser, once per week for 2 or 5 weeks, and were sacrificed at day 14 for micro-CT analysis and at day 35 for biomechanical test (n=10/group at each time point). (**B**) Temporal NIR-ICG images at 0 hour and 24 hours after ICG injection in 35 days post-fracture (n=10/group at each time point). (**C**) Quantitative analysis of draining lymphatic clearance in (**B**). (**D**) The gross anatomy of isolated PLNs at day 35 after fracture (top). Temporal ultrasound image of PLNs at day 35 post-fracture (n=10/group at each time point, bottom). Scale bars, 1mm. (**E**) Quantitative analysis of the PLN volume in (**D**). (**F**) Representative photographs of hindlimb at day 2 post-fracture (n=10/group). Scale bars, 1cm. (**G**) The relative swelling ratio of mouse hindlimb in 7 days after fracture (n=10/group). (**H**) Analysis of PWTL at day 1 (n=10/group). (**I**) Representative Micro-CT images (cross-section) of tibial fracture healing at day 14 post-fracture. (**J**) Quantification of BV/TV, Tb.N, Tb.Th and Tb.Sp (n=10/group). (**K**) Representative histomorphological images of ABH-stained sections at day 14 post-fracture. Red asterisk indicates fiber, Orange asterisk indicates cartilage, and black asterisk woven bone. The bottom images are higher magnifications of the regions boxed in black in the corresponding image above (n>8/group). Scale bars, 1 mm, 200 mm. (**L**) Callus composition in (**K**) were analyzed. (**M**) Analysis of biomechanical testing (n=8/group). Data are means±SD. *P<0.05; **P<0.01; ***P< 0.001; *NS*, not significant. In **C, E** and **G**, analysis of variance in repeated measurement design followed the simple effect test. In **H**, **J**, **L** and **M**, two-sided Student`s t-test.

**Figure 3:**
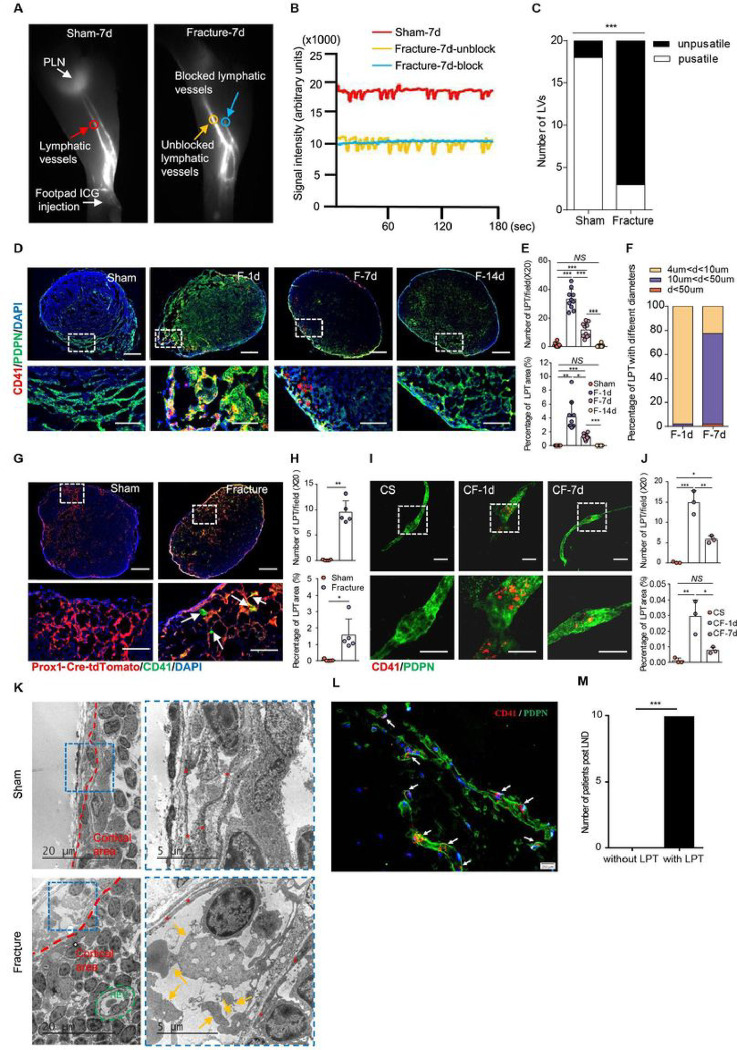
Platelets aggregation within the lymphatic vessels and subcapsular sinus of lymph nodes blocks lymphatic drainage (**A**) Representative NIR-ICG images showing draining LVs’ morphology and ROIs where the lymphatic pulses were measured (red circle indicates ROI at LVs from sham hindlimb, yellow and blue circle indicates ROI at LVs from fractured hindlimb, n=10/group at each time point). The interrupted ICG signal in the fractured hindlimb at day 7, marked by a blue circle, is defined as a blocked LV. (**B**) Lymphatic pulse corresponding to ICG signals in (**A**) at day 7. (**C**) The number of LVs with or without lymphatic pulse at day 7. *P < 0.05 vs sham by Fisher’s Exact Test. (**D**) Representative immunofluorescence staining on PLNs using anti-CD41 antibody to label platelet (red) and anti-podoplanin antibody for LVs (green). The bottom images are higher magnifications of the regions boxed in white in the corresponding image above (n>8/group). Scale bars, 200 mm, 50 mm. (**E**) Quantitative analysis of LPT number per field (×20) and LPT coverage area percentage. (**F**) Quantitative analysis of percentage of LPT with different diameters. (**G**) Representative immunofluorescence staining by anti-CD41 antibody (green) on PLNs from 7-week-old Prox1-cre-tgTomato mouse after surgery (n=5/group). The bottom images are higher magnifications of the regions boxed in white in the corresponding image above. Scale bars, 200 mm, 50 mm. (**H**) Quantitative analysis of LPT number per field (×20) and LPT coverage area percentage in (**G**). (**I**) Whole mount immunofluorescence staining at tail skin of tail fracture model (n=3/group) by anti-CD41 antibody (red) and anti-podoplanin antibody (green). The bottom images are higher magnifications of the regions boxed in white in the corresponding image above (n=3/group). Scale bar, 50 mm. (**J**) Quantitative analysis of LPT number per field (×20) and LPT coverage area percentage in (**I**). (**K**) Representative ultrastructural images of PLNs isolated from sham and fractured mice (n=3/group) at day 7 post-surgery by electron microscopy. Lymphatic endothelial cells are marked by red asterisks, while platelets are marked by orange arrows. SS in red color indicates subcapsular sinus, CA in red indicates Cortical area, HEV in green color indicates high endothelial venules. (**L-M**) Evidence for LPT widely existence in human LNs. (**L**) Representative immunofluorescence staining on dissected LNs from LND patients, using anti-CD41 antibody to label platelet (red) and anti-podoplanin antibody for LVs (green). Scale bar, 200 mm. (**M**) Number of LND patients with LPT in (**L**), n=10 patients. Data are means±SD. *P < 0.05; **P<0.01; ***P < 0.001; *NS*, not significant. In **E** and **F**, n=8 in sham group, n=9 in fracture-1d group, n=10 in fracture-7d and fracture-14d group, One way ANOVA. In **H**, n=5/group, two-sided Student`s t-test. In **J**, n=3/group, One way ANOVA.

Enlarged superficial LVs and draining lymph nodes were found in limbs with healed bone fractures, while reduced draining lymph nodes were seen in the majority of patients with non-union fractures^14,15^. Theses findings indicate that bone fracture affects the lymphatic system. However, the effect of proper lymphatic drainage on fracture healing is unclear. VEGFC/VEGFR3 signaling pathway is critical for lymphangiogenesis and LV drainage function^16^. To study the effect of promoting lymphatic drainage on fracture healing, we treated our mouse models of tibial fracture with recombinant human (rh) VEGF-C (Cys156Ser), which is specific to promote lymphangiogenesis and lymph flow^17^, or with vehicle at the time of surgery, and examined histomorphometry by micro-CT and callus composition by Alcian Blue-Hematoxylin/Orange G (ABH/OG) -staining at day 14. We also assessed bone quality by biomechanical testing at day 35 (Fig. 2A). VEGF-C therapy significantly increased lymphatic clearance at day 1 and at day 7 and PLN volume during the entire process of fracture healing (Fig. 2B–E). Furthermore, VEGF-C significantly ameliorated soft tissue swelling at day 1 and at day 3 post-fracture, and increased PWTL at day 1 post-fracture compared to the vehicle-treated group, respectively (Fig. 3F–H). Notably, rhVEGF-C treatment significantly increased BV/TV, Tb.N, and decreased Tb.Sp of the fracture callus at day 14, compared to the vehicle-treated group (Fig. 2I–J). ABH-staining and histomorphological analysis indicated that rhVEGF-C-treated group had significantly reduced cartilage and increased woven bone compared to the vehicle-treated group (Fig. 2K–L). Compared to the vehicle-treated group, VEGF-C also improved maximum torque, maximum flexural rigidity and fracture energy at day 35 (Fig. 2M). These results indicate that stimulation of lymphatic drainage promotes bone repair.

### Platelets aggregation within the lymphatic vessels and subcapsular sinus of lymph nodes blocks lymphatic drainage

Since sufficient lymphatic drainage is crucial for bone repair, we further investigated the underlying mechanism of LDI at the first 14 days post-fracture. Elias *et al*. reported that erythrolysates suppress lymphatic pumping^18^. However, few red blood cells are observed in the lymphatic system over 4 hours after subarachnoid hemorrhage^19^. In addition, inflammation is another well-recognized pathological factor that negatively influences lymphatic contractions, but inflammation is dramatically decreased by the 3rd day after fracture^20,21^. The classic notion that LDI is caused by inflammatory factors and erythrolysate cannot explain why decreased lymphatic drainage persistent for 14 consecutive days as observed here in this study, suggesting that another critical pathogenesis leads to LDI.

On day 7 after fracture initiation, NIR-ICG lymphatic imaging showed that 17 out of 20 LVs among the fracture group had interrupted ICG signal as evidenced by a lack of pulse at the distal part (Fig. 3A–C, Supplemental Movie 1). Platelets are anucleated blood cells that are 2–4 μm in diameter. They have a short lifespan, circulating in blood for only 7–10 days^22^. The inner diameter of lymphatic vessels is greater than 100 μm^23^, thus providing an anatomical opportunity for the entry of platelets. Based on well recognized deep vein thrombosis after fracture, we hypothesized that fracture-induced bleeding and thrombosis may activate and aggregate platelets, resulting in an accumulation of platelets within the LVs, therefore blocking lymph flow. We used an anti-CD41 antibody to label platelets and an anti-podoplanin (PDPN) antibody to visualize LVs. Immunostaining of PLNs showed a large number of CD41^+^ platelets that aggregated at PDPN^+^ subcapsular and paracortical areas in the fracture group. On day 1 after fracture initiation, large amounts of scattered platelets were observed in the LNs. On day 7, the number of individual platelets decreased and a large extent of platelet thrombosis within the LVs was formed. We referred to this platelet thrombosis as Lymphatic Platelet Thrombosis (LPT). On day 14, few LPT were found in the LNs (Fig. 3D). Similar LPT was detected in inguinal and iliac LNs (Supplementary Fig. 1). Both the number and percentage area covered by CD41^+^ platelets in the fracture group on day 1 and day 7 after fracture initiation were significantly greater than those of the sham group and fracture group on day 14 (Fig. 3E). The number and percentage area covered by CD41^+^ platelets in the fracture group on day 1 were significantly greater than those of fracture group on day 7 (Fig. 3E). The LPT was classified into three categories based on its various diameters. On day 1 after fracture initiation, small LPT with diameter less than 10μm accounts most. While LPT with longer diameter, ranged from 10 to 50μm, accounts most on day 7 after fracture initiation (Fig. 3F). In addition, we performed tibial fracture surgery in Prox1-Cre-tdTomato mice, a reporter strain that allowed us to observe Prox1^+^ LVs. Immunostaining of PLNs also identified a number of CD41^+^ platelet-derived clots in Prox1^+^ subcapsular and paracortical areas in the fracture group on day 7 post-surgery, compared to no such clots in the sham group (Fig. 3G). The number and percentage area covered by CD41^+^ platelets in the fracture group on day 7 were greater than those of the sham group (Fig. 3H). Subsequently, to further determine whether platelets were drained by LVs to PLNs, we performed whole-mount immunofluorescence staining using an anti-CD41 antibody and an anti-PDPN antibody on caudal skin after caudal vertebra fracture or sham fracture. We found CD41^+^ platelet aggregation in PDPN^+^ draining LVs in the fracture group, while no LPTs were observed in the sham group (Fig. 3I–J, Supplemental Movie 2–3). Ultrastructural analyses showed that subcapsular sinus dilated in areas where large amounts of activated and degranulated platelets were aggregated (Fig. 3K). To provide more confident evidence of widely existing LPT in bleeding condition, we collected 10 dissected LNs from patients with Lymphadenectomy (LND) and performed immunostaining. All LNs from LND patients showed large amount of LPT existing (Fig. 3L–M). These results identified that a large extent and high incidence of LPT formed in draining LVs and LNs during the early phase of fracture healing.

### Lymphatic platelet thrombolysis promotes fracture healing by unblocking lymphatic drainage

**Figure 4:**
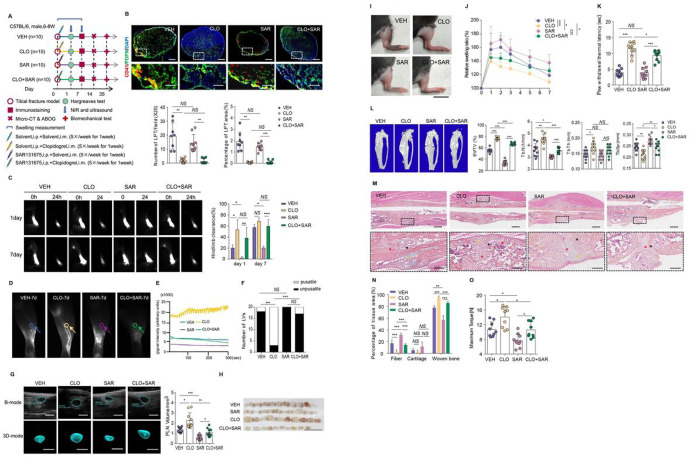
Lymphatic platelet thrombolysis promotes fracture healing by rejuvenating lymphatic drainage (**A**) Scheme of experimental procedure for Clopidogrel and VEGFR3 inhibitor intervention. After fracture, mice were treated with solvent, Clopidogrel, SAR131675, or Clopidogrel + SAR131675, once a day for 7 days, and were sacrificed respectively at day 7, 14 and 35 (n=10/group at each time point). (**B**) Immunofluorescence staining and quantitative analysis as Figure 4E. The bottom images are higher magnifications of the regions boxed in white in the corresponding image above. Scale bars, 200 mm, 50 mm. (**C**) Representative NIR-ICG images at hindlimb after ICG injection at day 1 and day 7 post-fracture and Quantitative analysis of draining lymphatic clearance (n=10/group at each time point). (**D-E**) Representative NIR-ICG images showing ICG signals in a draining LV at day 7 post-fracture. (**F**) Number of LVs with or without lymphatic pulse in (**D**) (n=20/group,*P < 0.05 by Pearson’schi-squared test). (**G**) Ultrasound images andQuantification of PLNs at day 7 post fracture (n=10 per group). Scale bars, 1mm. (**H**) The gross anatomy of isolated PLNs. Scale bars, 1 cm. (**I**) Representative photographs of hindlimb at day 2 post-fracture (n=10/group). Scale bars, 1 cm. (**J**) The relative swelling ratio of mouse hindlimb in 7 days after fracture (n=10/group, analysis of variance in repeated measurement design followed the simple effect test). (**K**) Analysis of PWTL at day 1 (n=10/group). (L) Representative Micro-CT images and quantificative analysis of callus at day 14 post-fracture (n=10/group). (**M**) Representative histomorphological images of ABH-stained sections at day 14 post-fracture. Red asterisk indicates fiber, Orange asterisk indicates cartilage, and black asterisk woven bone. The bottom images are higher magnifications of the regions boxed in black in the corresponding image above (n>8/group). Scale bars, 1 mm, 200 mm. (**N**) Callus composition in (**M**) were analyzed. (**O**) Analysis of biomechanical testing (n=9 in VEH, SAR and SAR+CLO group, n=10 in CLO group, One way ANOVA). In **B**, **C**, **G**, **K**, **L**, **N** and **O**, Values are mean±SD, One way ANOVA. *P < 0.05; **P<0.01; ***P < 0.001; *NS*, not significant.

Although fracture-induced LPT was definitely identified in draining LNs and LVs for more than 7 days, it can’t prove that LPT limits fracture healing via blocking lymphatic drainage. Clopidogrel, a P2Y_12_ antagonist, interferes with platelet activation mediated by ADP, inhibits platelet aggregation, and is widely treated for cardiovascular and cerebrovascular diseases^24^. We employed a low dose of Clopidogrel at the 4-hour post-fracture to eliminate LPT. To determine LPT inhibits fracture healing via lymphatic drainage, we designed 4 groups of mice, e.g. fractured mice treated with 1) vehicle (VEH), 2) Clopidogrel (CLO), 3) VEGFR3 inhibitor (SAR), and 4) Clopidogrel + VEGFR3 inhibitor (CLO + SAR), as a rescue experiment (Fig. 4A).

On day 7 post-fracture, compared to vehicle-treated mice, the number and percentage area covered by CD41 + platelets in CLO group were significantly lower and has no significant difference with SAR group (Fig. 4B). These data indicate that Clopidogrel induces lymphatic platelet thrombolysis. Compared to VEH group, CLO group significantly increased lymphatic clearance at day 1 post-fracture, the number of lymphatic pulses and PLN volumes at day 7 post-fracture, while SAR group shows the opposite trends, which can be rescued by CLO + SAR treatment (Fig. 4C–H, Supplemental Movie 4). These data indicate that Clopidogrel induced lymphatic platelet thrombolysis promote lymphatic draining function. Compared to VEH group, CLO group significantly ameliorated the swelling of soft tissue within 7 days post-fracture, increased PWTL at day 1 post-fracture (Fig. 4I–K). CLO group also significantly increased BV/TV, Tb.N and Tb.Th of the fracture callus by micro-CT and woven bone by ABH-staining at day 14 post-fracture and maximum torque at day 35 post-fracture (Fig. 4L–O). Compared to VEH group, however, SAR group showed increased soft tissue condition, worse histomorphometry, histomorphology and biomechanical property of callus, which was prevented by CLO + SAR dual therapy (Fig. 4L–O). In a word, these results demonstrated that eliminating LPT improves fracture healing by unblocking lymphatic drainage.

### Lymphatic drainage supports osteoblast survival and BMSC proliferation

Fracture healing is a complex and well-orchestrated physiological process including hematoma formation and inflammation, fibrovascular growth, bone formation and remodeling^25^. Multiple cell types and molecules are involved in each stage^25^. Hence, lymphatic drainage affect various stages of bone healing probably via different mechanisms. In this study, we focused on investigating the mechanism of lymphatic drainage-regulated fracture healing at the early stage due to the following reasons. (1) Hematoma and inflammation, generally lasting for about 3 days, are the initiating and foremost important stages of fracture healing^25,26^. Uncleared hematoma or dysregulated inflammation increases nonunion and delayed union rates^27,28^. (2) Lymphatic platelet thrombolysis restored lymphatic draining function most significantly at the early stage of fracture healing (Fig. 4C). At the early stage of fracture healing, committed OBs resides in the periosteum proliferate, migration and directly differentiate for intramembranous ossification, likewise, undifferentiated BMSCs are recruited to the injured sites, proliferate and differentiate for later endochondral ossification^29,30^. The absence of intramembranous and endochondral ossification will cease bone repair if the fracture gaps are unsuccessfully birdged^29^.

**Figure 5:**
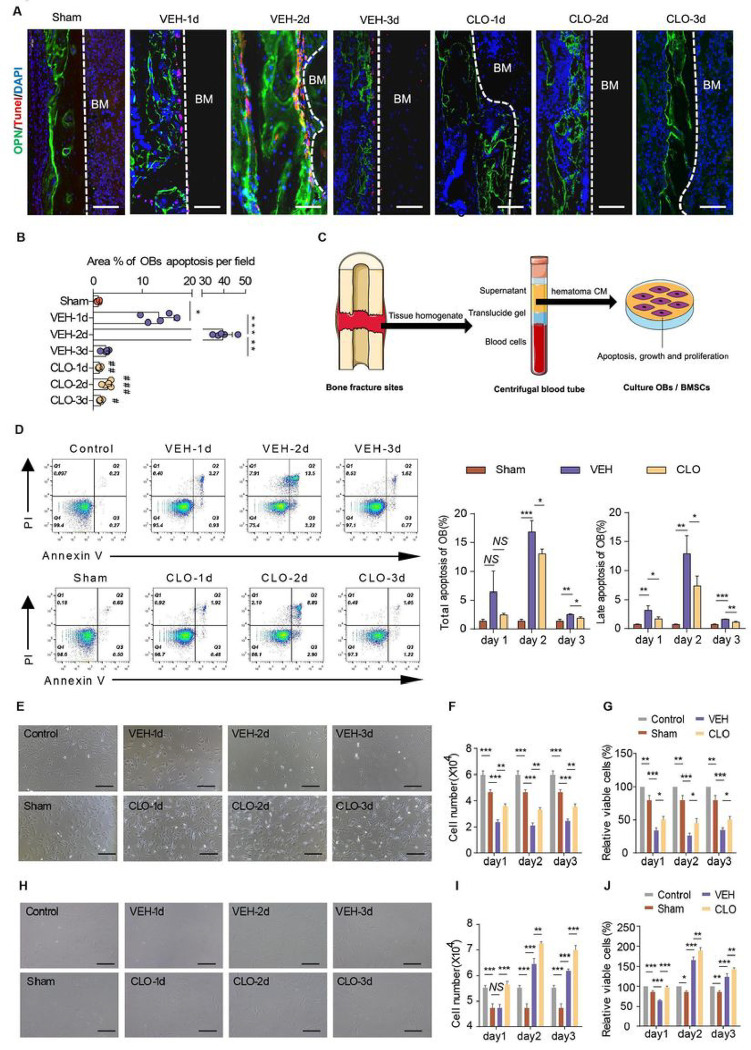
Lymphatic drainage supports osteoblast survival and BMSC proliferation (**A**) Representative immunofluorescence staining on fracture sides using anti-OPN antibody to label OBs (green) and anti-tunel antibody for apoptosis (red). Scale bars, 50 um. (**B**) Quantitative analysis of OBs apoptosis at fracture sides. (**C**) Scheme of experimental procedure in vitro for OBs and BMSCs treated by bone marrow or hematoma CM. Bone marrow or/and hematoma of fractured rats were collected to generate hematoma CM. Rat BMSCs and OBswere culturedwith hematomaCM for 24hand subjected to growth and proliferation analyses. (**D**) Hematoma CM intervened rat OBs for 1h and evaluated OBs apoptosis by flow cytometry with FITC-Annexin V and PI double staining. (**E**) Rat OBs were cultured with hematoma CM for 1h and observe the growth under a microscope. (**F**) The cell count of rat OBs with hematomaCM for 24h. (**G**) Rat OBs were cultured with hematoma CM for 24h and evaluated the cell proliferation using a CCK8 kit. (**H**) The cell growth of rat BMSCs, as in (**D**). (**I**) The cell count of rat BMSCs, as in (**F**). (**J**) The cell proliferation rat BMSCs, as in (**G**). In **B** and**D**, n=5/group. In **F-G** and **I-J**, n =3 wells in control, sham, VEH and CLO group. One way ANOVA. *P < 0.05; **P<0.01; ***P < 0.001; *NS*, not significant.

Fracture-induced osteocyte necrosis are acknowledged^30^, while fracture-induced OB death is rare to reported. We used osteopontin (OPN) to label OBs and TUNEL to label cell apoptosis and observed a large amount of OB apoptosis at day 1–3 post-fracture in vehicle-treated group, while OB apoptosis significantly decreased in Clopidogrel-treated group (Fig. 5A–B). BMSCs are a subset of stromal cells, present in the bone marrow at low frequency (less than 0.01% of the overall mononucleated cells in bone marrow) and capable of differentiation into bone and cartilage^25,26^. BMSCs lack specific and unique markers and a generic system to in vivo trace endogenous BMSCs during fracture has not developed yet. Hence, researchers were limited to directly observe the phenotypic changes of BMSC in vivo by histological morphology.

The hematoma at the fracture sides constitutes the early healing microenvironment, which was named after hematoma niche^26^. To determine whether lymphatic drainage-modulated hematoma niche supported OBs and BMSCs, we extracted the bioactive components of rats’ bone marrow/hematoma from sham group (without tibial fracture), vehicle-treated and Clopidogrel-treated group (with tibial fracture) respectively on day 1–3 post-surgery, generate CM simulating hematoma niche to culture OBs and BMSCs for 24h, and observe their apoptosis, growth and proliferation in vitro (Fig. 5C). We firstly applied ALP staining to identify rat caldaria-derived osteoblasts (Supplementary Fig. 2). Rat OBs were respectively cultured in DMEM/F-12 culture medium containing 10% FBS without hematoma CM (labeled as control group) and different hematoma CM for 1 hour and detected OB apoptosis by flow cytometry. Hematoma CM derived from fractured rats treated with vehicle significantly induced OBs apoptosis, while hematoma CM derived from fractured rats treated with Clopidogrel decreased the percentage of OB apoptosis (Fig. 5D). This result indicate that OBs treated with simplified extracted CM in vitro replicated OB apoptosis of histological morphology and suggest potentially applicable and scientific method of simulating hematoma niche to measure BMSCs. Cultured OBs in all experiment groups adhered to the plastic dish and demonstrated a triangular or spindle-like shape. We used hematoma CM to intervene OBs for 1 hour, compared to sham and Clopidogrel-treated group, the number of contracted and suspended OBs with less pseudopod are significantly increased in vehicle-treated group (Fig. 5E). Then OBs in various dishes were harvested and counted, the number of OBs in vehicle-treated group significantly decreased when compared with sham group and the number of OBs in Clopidogrel-treated group slightly increased when compared with VEH group (Fig. 5F). We also measured cell proliferation by a CCK-8 assay (Fig. 5G), which showed a consist trend as in Fig. 5F.

**Figure 6:**
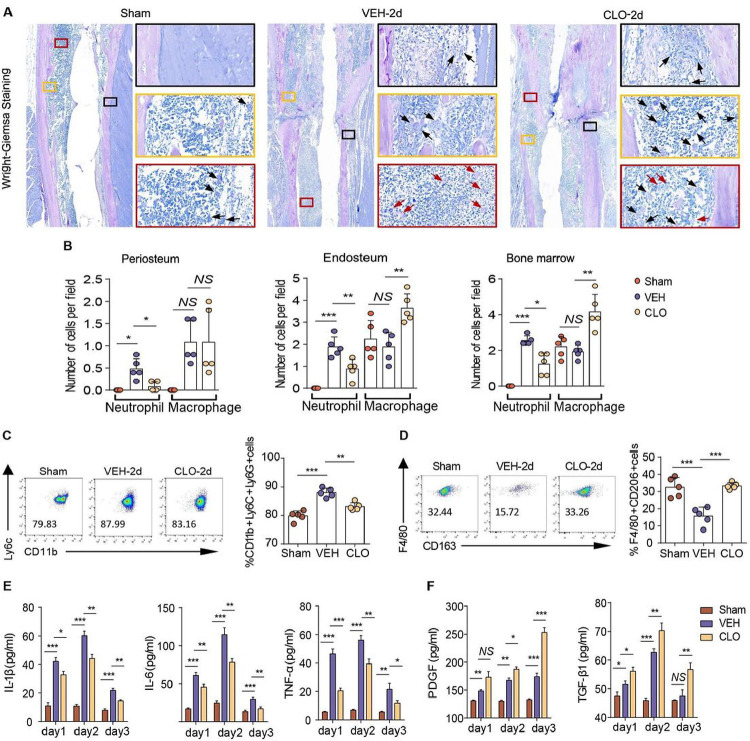
Lymphatic drainage immunomodulates hematoma niche (**A**) Representative Wright and Giemsa stain of mouse tibia on day 2 post-surgery. Scale bars, 50 um. (**B**) Quantitative analysis of neutrophil and macrophage of endosteum, periosteum and BM in (**A**). (**C**) Quantitative analysis of neutrophil in hematoma niche by flow cytometry. (**D**) Quantitative analysis of macrophage in hematoma niche by flow cytometry. (**E**) The concentration of pro-inflammatory cytokines for hematoma homogenate on days 1,2 and 3 post-sugery by ELISA. (**F**) The concentration of growth factors for hematoma homogenate on days 1,2 and 3 post-sugery by ELISA. In **B**, n=5/group. In **C-D** and **E-F**, n =3 wells in control, sham, VEH and CLO group. One way ANOVA. *P < 0.05; **P<0.01; ***P < 0.001; *NS*, not significant.

To identification of rat BMSC isolation, we firstly examined the cellular markers and differentiation capability of isolated cells. Bone marrow-derived cells expressed rat BMSC markers by flow cytometry analysis and differentiate into OBs, osteocytes and chondrocytes in vitro (Supplementary Fig. 3). BMSCs at all groups adhere to the plastic dish and demonstrated a fibroblast-like or spindle-like shape (Fig. 6H). Both the cell number count and CCK-8 assay showed that BMSCs in Clopidogrel-treated group were more than in vehicle-treated group (Fig. 5I–J). These data demonstrate that (1) OB apoptosis occurs at the inflammatory and hematoma phases of fracture healing. (2) Up-regulated lymphatic draining function supports OB survival and BMSC proliferation at hematoma niche.

### Lymphatic drainage immunomodulates hematoma niche

When a bone is injured, diverse immune cells are recruited to the site of injury and secreted multiple cytokines, such as pro-inflammatory and growth factors, to ensure successful fracture healing^32^. Pro-inflammatory factors (TNF-a, IL-1 and IL-6) and growth factors (TGFβ1 and PDGF) are vital ligands of OB apoptosis and BMSC proliferation^21,33^. Therefore, we questioned whether lymphatic drainage immunomodulated these immune cells, those paracrine secrete cytokines to influence surrounding OB apoptosis and BMSC proliferation. Wright and Giemsa Stain, flow cytometry and enzyme-linked immunosorbnent assay (ELISA) of pro-inflammatory and growth factors were applied to observe the spatial-temporal changes of hematoma niche.

On day 2 post-sugery, Wright and Giemsa Stain on tibial paraffin sections showed that neutrophils significantly increased in the peiosteum, endosteum and bone marrow of vehicle-treated mice compared with sham mice, while decreased in Clopidogrel-treated mice compared with vehicle-treated mice. Clopidogrel-treated mice significantly have more monocytes and macrophages in the endosteum and bone marrow than vehicle-treated mice (Fig. 6A–B). Flow cytometry of neutrophils and M2-like macropahes showed that the hematoma niche of Clopidogrel-treated mice significantly have less neutrophils and more M2 macrophages than vehicle-treated mice (Fig. 6C–D). On day 1 and 3 post-fracture, the hematoma niche of Clopidogrel-treated mice shows the same trend as compared with vehicle-treated mice (Supplementary Fig. 4–5). Moreover, we utilized the ELISA kits to examine levels of pro-inflammatory and growth factors in supernatant of callus hematoma extracts from vehicle or Clopidogrel-treated rats. Lower levels pro-inflammatory factors and higher levels of growth factors were detected in Clopidogrel-treated group than vehicle-treated group (Fig. 6E–F). These results elucidate that unblocked lymphatic drainage decreased neutrophils and increased M2-like macrophages of hematoma niche to support OB and BMSC.

### Draining lymphatic fluid inhibit OB and BMSC

**Figure 7:**
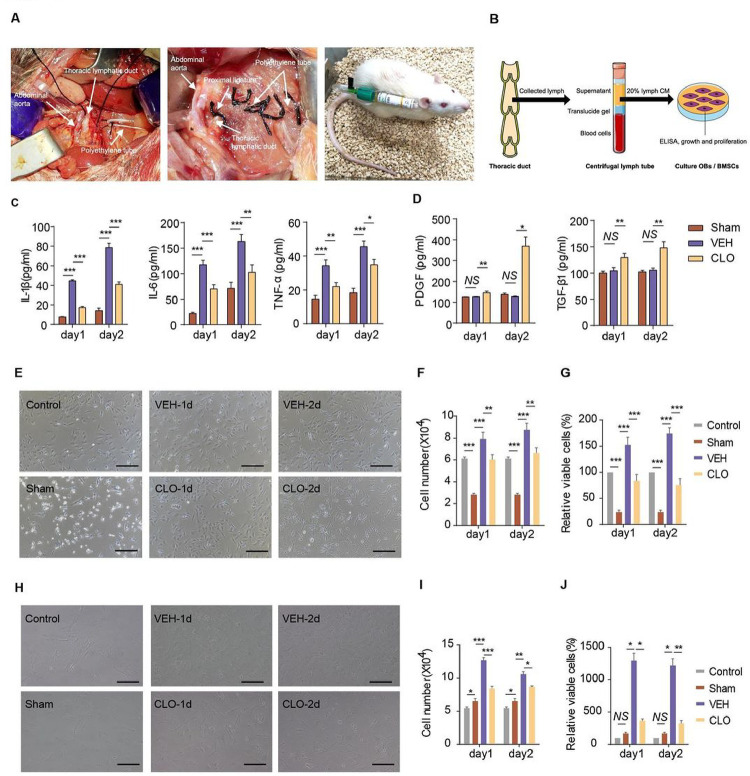
Draining lymphatic fluid inhibit OB and BMSC (**A**) Surgical procedures of thoracic lymph duct cannulation. Mesenteric lymphatic duct was retracted at first and then cannulated. Lymph was collected by anticoagulant tube. (**B**) Scheme of experimental procedure in vitro for OBs and BMSCs treated by 20% lymph CM. Thoracic duct lymph from sham and fractured rats treated with vehicle or Clopidogrel were collected to generate lymph CM. Rat BMSCs and OBs were cultured with 20% lymph CM for 24h and subjected to Elisa, growth and proliferation analyses. (**C**) The concentration of pro-inflammatory cytokines for 20% lymph CM on days 1,2 and 3 post-sugery by ELISA. (**D**) The concentration of growth factors for 20% lymph on days 1,2 and 3 post-sugery by ELISA. (**E**) Rat OBswere culturedwith 20% lymphCM for 24h and observe the growth under a microscope. (**F**) The cell count of rat OBs with 20% of CM for 24h. (**G**) Rat OBs were cultured with 20% lymph CM for 24h and evaluated the cell proliferation using a CCK8 kit. (**H**) The cell growth of rat BMSCs, as in (E). (**I**) The cell count of rat BMSCs, as in (**F**). (**J**) The cell proliferation of rat BMSCs, as in (**G**). In **C-D**, **F-G**, **I-J**, n =3 wells in control, sham, VEH and CLO group. One way ANOVA. *P < 0.05; **P<0.01; ***P < 0.001; *NS*, not significant.

With abundance of fibrin network, extracellular matrix (ECM) and various cytokines, the hematoma derived-tissue fluid constitutes the micro-environment of BMSCs and OBs^26^. These molecular components are transported into the systemic circulation through both venous and lymphatic capillaries. To distinguish the different transport function between the blood and lymphatic circulation system after fracture, we first applied cannulation of thoracic lymph ducts and femoral vein within the sham rats (rats without tibial fracture), vehicle-treated and Clopidogrel-treated rats (rats with tibial fracture) to collect the draining lymph (Fig. 7A) and venous blood. Then, collected draining lymph and venous blood were generated as different CM, respectively, and the concentration of inflammatory and growth factors in them was examined by ELISA. The effects of lymph and venous blood-derived CM on growth and proliferation of BMSCs and OBs were examined in vitro cultures (Fig. 7B and Supplementary Fig. 7A). At the first 2-hours post-surgery, the volume of collected draining lymph is most in sham group and least in vehicle-treated group (Supplementary Fig. 6A–B). This data is also a supporting evidence that lymphatic platelet thrombolysis unblocks lymphatic drainage. Due to technical issue (blockage of the polyethylene tubing) we could only collect draining lymph within 48h after the cannulation of thoracic duct (Supplementary Fig. 6C). Our preliminary data demonstrated that high concentration of lymph CM kills OBs and BMSCs in vitro and the optimal concentration of lymph CM was 20%, which was used in the subsequent experiments (Supplementary Fig. 6D).

Lymph CM from Clopidogrel-treated group contains lower levels of pro-inflammatory factors and higher levels of growth factors than vehicle-treated group (Fig. 7C–D). However, lymph CM from Clopidogrel-treated group inhibits OB and BMSC growth compared with lymph CM from vehicle group based on the results of cell growth, number and CCK8 assay (Fig. 7E–J). In addition, compared to vehicle-treated group, venous blood CM from Clopidogrel-treated group support OB and BMSC perhaps due to lower pro-inflammatory factors and higher growth factors (In Supplementary Fig. 7). These results demonstrate draining lymphatic fluid inhibit OB and BMSC and suggest that bone fracture induces some bioactive substances at fracture sides, those are (1) detrimental to OBs and BMSCs and (2) mainly transport by unblocked lymphatic capillaries rather than blood capillaries.

### Lymphatic vessels transport DAMPs from hematoma niche

**Figure 8:**
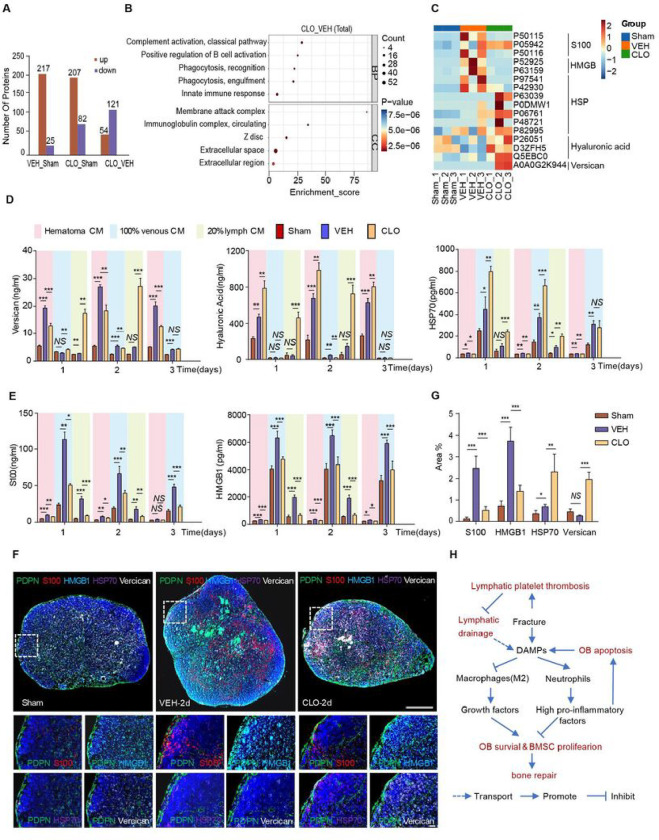
Lymphatic vessels transport DAMPs from hematoma niche (**A-C**) Proteomics analysis of thoracic duct lymph from sham and fractured rats treated with vehicle or Clopidogrel on day 1 post-surgery. (**A**) The number of increased and decreased proteins in thoracic duct lymph. (**B**) Top 10 GO biological process and celluar component enriched with total proteins in CLO compared to VEH. (**C**) Heatmap of DAMPs-associated proteins in thoracic duct lymph. (**D**) The concentration of HMW-DAMPs on days 1, 2 and 3 post-sugery by ELISA. (**E**) The concentration of LMW-DAMPs on days 1, 2 and 3 post-sugery by ELISA. (**F**) Representative mIHC images of PLN showing the positive expression of DAMPs. Upper scale bars: 500μm, lower scale bar: 50um. (**G**) The percentage of DAMPs+ coverage area was quantified by Image J. (**H**) Summary chart and the keywords of novel findings are underlined with red color. In **A-E**, n =3/group. In **G**, n=4 in sham group and n=8 in Vehicle and Clopidogrel-treated group. One way ANOVA. *P < 0.05; **P<0.01; ***P < 0.001; *NS*, not significant.

To identify harmful bioactive substances present in lymph that was transported from fractured site, we examined the protein profile of lymph collected from thoracic duct of rats by proteomics. Compared to sham group (rats without tibial fracture), the numbers of increased proteins at both vehicle-treated and Clopidogrel-treated groups (rats with tibial fracture) were significant more than decreased proteins (Fig. 8A), indicating that LVs are capable of transporting proteins expressed at fracture sides. A total of 165 different proteins were identified in lymph from vehicle-treated and Clopidogrel-treated groups and were analyzed by Gene Ontology (GO). Top 5 GO biological processes contained proteins that are enriched in innate immune response, complement activation, and phagocytosis, most of which are localized in extracellular space and cytoplasm (Fig. 8B). We further identified that 3 out of 12 enriched proteins in innate immune response, namely P50115 (Protein S100-A8, S100a8), P50116 (Protein S100-A9, S100a9) and P52925 (High mobility group protein B2, Hmgb2), are belonged to damage associated molecular patterns (DAMPs). DAMPs, including versican, low molecular weight hyaluronan, S100 proteins, heat shock proteins and high mobility group proteins, are endogenous danger molecules that are immediately released from the extracellular and intracellular space following tissue injury or cell death^34^. DAMPs activate the innate immune system to initiate and amplify inflammatory response by interacting with pattern recognition receptors^34,35^. Uncontrolled DAMPs in local inflammatory response aggravate to delayed fracture healing, sepsis, a sterile systemic inflammatory response syndrome, and multiple organ failure^35,36^. Heat map of DAMPs revealed that S100a8, S100a9, HMGB2, P63159 (High mobility group protein B1, HMGB1), P97541(Heat shock protein beta-6, Hspb6) and P42930 (Heat shock protein beta-1, Hspb1) were significantly increased in lymph from vehicle-treated rats while other larger molecular weight of HSPs, HA metabolic related proteins and versican were significantly increased in lymph from Clopidogrel-treated rats (Fig. 8C). Thus, we hypothesized that unblocked LVs within or surround hematoma niche support OB and BMSC by transporting DAMPs.

To test our hypotheses and distinguish the different transport function of DAMPs between the venous and lymphatic system, we measured the concentrations of widely recognized DAMPs by ELISA in hematoma CM, 20% lymph CM and 100% venous CM of sham rats and fractured rats treated with vehicle or Clopidogrel. Versican, HA and HSP70 were classified as high molecular weight DAMPs (HMW-DAMPs) while S100 and HMGB1 were classified as low molecular weight of DAMPs (LMW-DAMPs) in Fig. 8D–E. For hematoma CM, compared to Sham group, the levels of both HMW-DAMPs and LMW-DAMPs were significantly increased in VEH group. Compared to VEH group, the levels of HMW-DAMPs (except for HA) and LMW-DAMPs were significantly reduced in CLO group. It indicate that Clopidogrel increased the accumulation of DAMPs at hematoma niche. For 20% lymph CM, compared to VEH group, the level of HMW-DAMPs significantly increased in CLO group while the level of LMW-DAMPs significantly decreased in CLO group. We suspected that unblocked LVs transported HMW-DAMPs broken the vicious cycle of high immune and inflammatory response and reduced generation and accumulation of LMW-DAMPs at the fracture sites. For 100% venous CM, compared to CLO group, the level of LMW-DAMPs significantly increased in VEH group probably due to higher immune and inflammatory responses of hematoma niche in VEH group. It indicate that unblocked LVs have an specific role in transporting HMW-DAMPs from hematoma niche. Rat thoracic lymph, the final end of lymphatic circulation, could not directly reflect the real time condition of local lymphatic draining of fractured hindlimb. We further utilized multiplex immunohistochemical (mIHC) to observe draining LNs of sham and fractured mice. Compared to vehicle-treated group, Clopidogrel-treated group showed significantly increased coverage area of Versican + and HSP70 + markers and decreased coverage area of S100 + and HMGB1 + markers (Fig. 8F–G). Based on theses findings, we concluded that (1) multiple DAMPs that are produced at fracture sites are drained/cleared via both LVs and venous capillaries; and (2) unblocked lymphatic capillaries are capable of transporting both HMW-DAMPs and LMW-DAMPs, while unblocked venous capillaries transport mainly LMW-DAMPs.

Although neutrophils are innate immune phagocytes that have a central role in immune defence via releasing of neutrophil extracellular traps (NETs) to kill or suppress fungal and bacterial proliferation^37^. Excess or uncleared NETs can also lead to the pathological changes, such as tissues damage, promoting M1-like macrophage activation to secrete inflammatory factors, vaso-occlusion^37^. Versican and hyaluronan are extracellular matrix (ECM) proteoglycans and interact with neutrophils to promote their adhesion, accumulation, and secrete inflammatory cytokines^35,38^. Extracellular HSPs are cellular necrosis products and also induce immune cells to secrete inflammatory cytokines through activating TLR2, TLR4, and CD91^34,39^. Relieving immune and inflammatory response is beneficial for anti-inflammatory M2-like macrophages to secrete growth factors to support OBs and BMSCs^32^. These suggests that bone fracture induced LPT blocks lymphatic drainage and limited bone regeneration while unblocked lymphatic drainage increased clearance of DAMPs, and decreased neutrophils and increased M2-like macrophages of hematoma niche to support OBs and BMSCs (Fig. 8H).

## Discussion

In this study, we found that lymphatic drainage at the fracture site dramatically deceased soon after fracture and then generally returned to normal level by 14th day post-fracture, and sufficient lymphatic drainage benefits fracture repair. We further identified that LPT limits fracture healing via blocking lymphatic draining function. At last, we found lymphatic platelet thrombolysis immunomodulated hematoma niche to ameliorate OB apoptosis and promote BMSC proliferation via promoting lymphatic transporting DAMPs.

### An early alarm for tissue repair disorder: LDI

Whether in textbooks or empirical therapy, scientists and clinicians mainly care about blood supply for tissue repair. Recent research reveals that LVs are involved in tissue regeneration, such as heart, intestine and bone, via different lymphangiocrine signals ^10,40–42^. Our study for first time demonstrated that lymphatic draining is essential and irreplaceable in bone repair via transporting of HMW-DAMPs. Blocked LVs fails to transport HMW-DAMPs, expands and prolong immune and inflammatory response, and inhibits bone repair. Therefore, except blood circulation, advanced age, fracture type, severity of soft tissue injury and smoking, LDI may be an independent risk factor for delayed union and nonunion.

Lacking of early warning approaches to prevent nonunion and delayed unnion in advance, when fractured patients are definitely diagnosed as nonunion or delayed union by plain radiographs, they have missed the best opportunity for intervention. Based the clinical researches by Szczesny, G and collaborator^14,15^, and our experimental results, employing NIR-ICG and ultrasound of draining LNs at the early phase of fracture may be a new and simple approaches to predict bone healing. Fractured patients with sustained LDI without enlarged draining LNs as long as more than 2 weeks post-fracture predicts the high risk of delayed nonunion and delayed union.

Emerging evidences indicate that inflammatory diseases, such as rheumatoid arthritis, osteoarthritis, Alzheimer’s disease and Parkinson’s disease are well-relieved by promoting lymphatic drainage or eliminating DAMPs^43–46^. What the relationship between lymphatic draining fucntion and clearance of DAMPs remains unknown. Our work also elucidates that lymphatic clearance of DAMPs is the underlying mechanism of targeting LVs to treat inflammatory diseases.

### Insight for lymphatic thrombosis

Unlike venous or arterial thrombosis, lymphatic thrombosis is generally considered to be rare and mainly occurs in cancer, infections, congestive heart failure, chronic edema and inflammation^47^. Its proposed pathological mechanism, similar to the Virchow triad characteristic of venous thrombosis, involved the release of thromboplastin substances from damaged lymphatic endothelium, resulting in a hypercoagulable milieu and chronic obstruction of lymphatic flow^47–49^. However, our data indicate that massive platelet thrombosis blocked all the draining lymphatics and lymph nodes for at least 14 days after fracture. Podoplanin, a well-recognized specific marker of lymphatic endothelial cells (LECs), can bind to platelet surface C-type lectin-like receptor 2 (CLEC-2) to activate platelets and form a thrombosis^50–51^. We also found that platelets tends to adhere to or cross the LEC wall (Data were not shown), suggesting that extravasated platelets are inclined to migrate to LECs and then activated to form LPT. Therefore, evidences above shows a high incidence of LPT, widely ignored by widely ignored by scientists and clinicians, during the early phase of fracture and hemorrhagic diseases.

Fracture induced vein thrombosis, breaking off the pulmonary artery, is commonly regarded as one critical cause of pulmonary embolism^52^. Based on the lymphatic circulation pathway, that LPT travels to the pulmonary circulation via the subclavian vein and inferior vena cava, might be another insidious and crucial pathogenesis of post-traumatic pulmonary embolism when no venous thrombosis are detected in clinic.

### Insight for antiplatelet therapy during the perioperative management

To decrease the risk of perioperative bleeding, Clopidogrel is recommended to discontinue at least 3–5 days before surgery^53^. High dose of administering Clopidogrel for long time also negatively affects bone health and fracture healing in mice^54,55^. However, many clinical studies also demonstrated that Clopidogrel did not increase perioperative bleeding, mortality^53, 56–57^. The withdrawal of clopidogrel are associated with increased postoperative complications, such as skin ulcers, myocardial ischemia, thromboembolic events and coronary stent thrombosis^53,58^. Therefore, it has been controversy surrounding the use of blood thinner. We demonstrate a low dose of Clopidogrel, about one tenth of the regular dose, could eliminate LPT to recover lymphatic drainage soon and improve fracture healing in mice. This work does not advocate patients with fracture for taking blood thinner, but supply a benefit evidence of antiplatelet therapy for the perioperative management of vascular events particularly in patients following coronary stenting. Circumstances alter cases, the risks of perioperative bleeding must be weighed against the thrombotic risks of Clopidogrel discontinuation.

Many clinical study mainly focus on the safety of antiplatelet therapy during the perioperative management and few studies concern the bone fractured healing indexes and perioperative complications, such as the union and delayed union rates, bone healing quality at 1–3 months post-fracture, soft tissue condition, post-traumatic limb edema. Contrast to long bone healing time for patients with fracture, we inferred lymphatic thrombolysis may ease early perioperative complications and decrease the risk and rate of fracture non-union and delayed union in clinic. In the future, high quality of clinical researches focus on early phase of bone healing and optimal antiplatelet agents for LPT during perioperative management should be carried out for guiding clinical practice.

### Limitations of the study

Our study has several technical and ethical limitations. Firstly, we optimized a protocol for extracting bioactive components from hematoma and use them to treated BMSCs to mimic micro-environment of fracture sites. However, this cannot substitute the actual and real-time changes of cellular micro-environment induced by fracture. Secondly, this research needs a direct confirmation of the occurrence of LPTs at the site of fractures. We have attempted to collect blood clot at fracture sides, venous blood and draining lymph from patients with bone fracture for treating human bone marrow stromal cells. However, none patients with lower limb fractures are willing to participate in this study. We have to collect LNs from lymphadenectomy patients as an indirect confirmation of the occurrence of LPTs. Lastly, low-molecular-weight hyaluronan (LMW-HA) was an endogenous activator of a TLR2-promoted immune and inflammatory response, whereas high-molecular-weight hyaluronan (HMW-HA) inhibits TLR2 signaling^59^.we can`t further distinguish the changes of LMW-HA or HMW-HA in CM due to a lack of a commercially available ELISA kits to measure LMW-HA and HMW-HA.

## MATERIALS AND METHODS

### Animals

6 ~ 8-week-old male C57BL/6 mice and SD rats were obtained from Shanghai Jessie Experimental Animal Co., Ltd. 6 ~ 8-week-old male transgenetic mice (Prox1-tdTomato) were purchased from the Jackson Laboratory^60^. All animal experiments described in this report were approved by Shanghai University of Traditional Chinese Medicine-Animal Ethics Committee (PZSHUTCM211101023). All animals were randomly assigned to each group and accommodate for 1 weeks before experimental procedures.

### Fracture model

We performed three types of fracture models in this study. (1) For open tibial fracture of mice, we used a scalpel blade to transect the right tibia carefully at the upper third without any injury to fibula and draining LVs behind the tibia^9^, and inserted a 0.45 (outer gauge)×16mm (inner gauge) intradermal needle (WEGO Holding Co., Ltd. Weihai, Shandong Province, China) through the anteromedial tibial plateau to access the medullary canal for intramedullary fixation^61^. While the sham group mice, a 15-mm skin incision was made over the anterior aspect of the right lower leg, and intradermal needle was inserted through the anteromedial tibial plateau to access the medullary canal without tibial fracture osteotomy. (2) For closed caudal fracture of mice, we open the caudal skin carefully, remove the last three segments of vertebrae and sew up the wound. While the sham group mice, we only open the caudal skin and sew up the wound without removing the last three segments of vertebrae. (3) For closed tibial fracture of rat and mice, to mimic real high energy trauma and perioperative period, we didn’t insert intramedullary pin into the tibia before establishing closed fracture model as previous literature described^62^. The femoral diaphysis was then fractured by a blunt guillotine driven by a dropped weight. While sham group is normal fed without any operation. All experimental animals were subjected to anesthetize via isoflurane inhalation (5% induction, 2.5% maintenance) during operation.

### Treatment

(1) For recombinant human VEGF-C protein treatment, Cys156Ser (Cat. No. 752-VC-025/CF, R&D Systems) was dissolved in sterile phosphate-buffered saline (PBS) solution and intramuscularly injected near the fracture site at 0.08 mg/kg of body weight for 2 weeks or 5 weeks (once a week) immediately after the tibial fracture was established in Fig. 2. The control group was given the same dose of immunoglobulin G (IgG), polyclonal Syrian hamster IgG (Cat. No. BP0087, BioXcell). (2) VEGFR3 tyrosine kinase inhibitor administration, SAR131675 (Cat. No. HY-15458, MedChemExpress, 250mg) was dissolved in a solvent, containing 2.5mL dimethyl sulfoxide, 15mL polyethylene glycol, 2.5mL Tween-80 and 30mL double distilled water. Then the dissolved SAR131675 (50 mg/kg of body weight) was intraperitoneally injected once per day for 2 weeks (5 days per week) immediately after tibial fracture established. The control group was given the same volume of solvent. (3) For Clopidogrel treatment, Clopidogrel (Cat. No. HY-15283, MedChemExpress) was dissolved in a solvent (2.5mL dimethyl sulfoxide, 15mL polyethylene glycol, 2.5mL Tween-80 and 30mL double distilled water), and intramuscularly injected near the right popliteal lymph node at 0.1mg/kg of body weight for 5 days (once per day). Clopidogrel was given at 4 hour after tibial fracture under the guide of ultrasound. The control group was given the same volume of solvent.

### Near-infrared indocyanine green (NIR-ICG) lymphatic imaging

After hair removal from the hind limbs, under isoflurane anesthetization, the mice were fixed on a hot-plate universal platform with a constant temperature of 37°C. The mice were subcutaneously injected with 5 μL 0.2 mg/ml ICG solution (Cat.No.17478–701-02, Akorn) into the footpads. Under an NIR laser (Changchun Laser Technology), ICG fluorescence of the afferent LVs from the injection site to the PLN were observed. The ICG clearance was assessed by calculating the percentage clearance of footpad region of interest (ROI) at 0 hour and 24 hours after ICG injection. The ICG signal was recorded continuously for 300 seconds by an Olympus microscope (exposure times 200 ms) and synthesized as NIR real time video. Images on lymphatic clearance and videos on lymphatic pulses were analyzed by Image J software as previously described^43^. Data analysis was performed in a blinded fashion with regard to group allocations.

### Ultrasound imaging

Under isoflurane anesthesia, after the hair removal of the hind limbs, the mice were fixed in a prone position on a universal hot plate platform with a constant temperature of 37°C. The PLNs were scanned, and the images were recorded by using an MX550D ultrasonic probe fixed on the 3D Motor Assembly, under Mouse Superficial tissue mode and 0.04 mm scanning layer thickness. The 3D reconstruction was established, and the maximum cross-sectional area in B mode and the volume of PLN in 3D mode was obtained by Vevo Lab 3.2.0 software. Data analysis was performed in a blinded fashion with regard to group allocations.

### Swelling measurement

The swelling of the fracture part was measured by our homemade swelling meter, based on an improvement of the plethysmometer paw volume meter test for rats^63^. Briefly, we kept the right hind limb below the level of knee joint completely immersed in a container filled with liquid. The volume change in the container before and after measurement was calculated as the swelling volume of the hind limb of the mouse. Each data was measured 3 times and averaged. Swelling degree=(measurement - baseline)/baseline *100%.

### Hargreaves test

For assessing thermal pain sensation in mice, the amount of time in a dynamic plantar tester (Cat.No. 37370, Ugo Basile,) that elicits a withdrawal response is termed as withdrawal latency. A longer withdrawal latency signifies a slower withdrawal response and vice versa^64^. After the mice were acclimated to the testing environment, 30% radiant heat was used to induce the withdrawal response. The paw withdrawal thermal latency (PWTL) value was recorded automatically by the instrument and was determined when the mice felt pain and raised their hind paw. Each mouse was tested five times. To obtain the average reaction time for every mouse, the lowest and the highest values were removed as outlying values and the remaining 3 values were averaged and recorded.

### Micro-Computed Tomography Analysis

The fractured tibias were harvested at day 14 post-fracture by careful dissection and removal of the intramedullary pin. The sample was fixed in 4% paraformaldehyde for 24 h, washed for 2 h, and soaked in 75% ethanol. The samples were scanned by high resolution μCT (SkyScan 1176, Bruker, Belgium), and the following parameters were used: 9 μm resolution, 0.5 mm aluminum filter, 70 kV voltage, and 142 μA current. All imaging and data were acquired by commercial software provided by the company. As previously described^65^, the major parameters for callus quantity and fracture healing included bone volume/total volume (BV/TV), trabecular number (Tb.N), trabecular thickness (Tb.Th), trabecular separation (Tb.Sp).

### Special staining and histomorphometric analysis

Harvested tibias were completely decalcified in 10% EDTA and embedded in paraffin. Paraffined specimens in midpoint of sagittalsection were selected and the cut at a thickness of 6 mm. We utilized two special staining of paraffin embedded mice tibial section. (1) ABH/OG staining was used to evaluate tibial callus on day 14 post-fracture. Sections were stained with Alcian blue/hematoxylin (ABH) and counterstained with eosin/orange G. All stained slices were then scanned with an Olympus VS-120 whole-slide imaging system. Quantitative analysis of the callus composition were measured using SlideViewer software (version 2013.3) in a blinded manner. The mean value of one magnification (×10) images of each tibia was calculated as one sample data. (2) Wright-Giemsa staining was applied to observe myeloid cells around tibial fracture sites on day 1–3 post-fracture. Slides were stained with Wright-Giemsa staining kit (Cat.No.C230511, BASO) according to the manufacturer’s protocol. All stained slices were then scanned with an Olympus VS-120 whole-slide imaging system. Quantitative analysis of the callus composition were measured using SlideViewer software (version 2013.3) in a blinded manner. The mean value of five magnification (×40) images of each tibia was calculated as one sample data.

### Biomechanical testing

The healed tibia of the right hind leg at day 35 post-fracture was harvested and sent for three-point bending tests by a standard materials testing machine (Model 5569; Instron Corp., Norwood, MA, USA) as previously described^66^. Primary fracture sides of each tested tibia were placed at a midpoint of two supports spaced 6 mm apart. The bending load was applied at the midpoint at a constant displacement rate of 1 mm/min until the sample fractured. Maximum torque, maximum flexural rigidity and fracture energy were calculated by a custom program (MATLAB; MathWorks Inc., Natick, MA, USA). All samples were blinded throughout preparation and testing.

### Immunofluorescence staining and histomorphometric analysis

(1) For immunofluorescence staining of PLNs, the harvested LNs were cleaned with PBS and photographed. For tissue processing, PLNs were fixed in 4% paraformaldehyde, dehydrated in a gradient sucrose solution, embedded in OCT and stored at −80°C. A cryostat (Leica, CM3050S) was then used to cut 7-μm-thick frozen sections. The primary antibodies for PLNs included syrian hamster monoclonal anti-podoplanin antibody (Cat.No.ab11936, Abcam, 1:1000) and rabbit monoclonal anti-CD41 antibody (Cat.No.ab134131, Abcam, 1:1000). The corresponding secondary antibodies were used as follows: Alexa Fluor 488 goat anti-hamster antibody (Cat. No. A-21110, Invitrogen, 1:200), Alexa Fluor 488/555 goat anti-rat antibody (Cat.No. 4416S/4417S, Cell Signaling Technology, 1:200). (2) For immunofluorescence staining of tibial paraffin section, the fractured tibias were harvested at day 1–3 post-fracture by careful dissection and removal of the intramedullary pin. The sample was fixed in 4% paraformaldehyde for 24 h, washed for 2 h, and completely decalcified in 10% EDTA and embedded in paraffin. Paraffined specimens were cut at a thickness of 6 mm and 3 levels (each level was cut 50 μm apart). The dewaxing tibial sections were immuno-stained tunel first using TUNEL BrightRed Apoptosis Detection Kit (Cat.No.A113–03, Vazyme, 1:1000) as manufacture’s instruction. Then tunel stained tibial sections were immunostained Osteopontin (OPN). The primary antibodies for tibias included anti-Osteopontin antibody (Cat.No.AF808, R&D systems, 1:1000) and the corresponding secondary antibodies is Alexa Fluor 488 donkey anti-goat antibody (Cat. No. abs20026, Absin, 1:200). Frozen PLNs and dewaxing tibial sections were blocked by 0.3% PBST with 5% bovine serum albumin for 1 h at room temperature, then incubated with primary antibodies overnight at 4°C. After washing with PBS, secondary antibodies were incubated for 2 h at room temperature. Finally, the sections were mounted with 4,6-diamidino-2-phenylindole (Cat.No. H-1200, Vectorlabs).

Images were acquired by using an Olympus VS120 microscope under a 20×objective. (1) The number of LPTs per magnification (×20) image in PLNs was by manual counting, both aggregated and scattered CD41 + platelets included. The coverage area ratio and the diameter of LPT was measured by ImageJ software. (2) The coverage area % of OB apoptosis per magnification (×20) image was measured by ImageJ software. The mean value of five magnification (×20) images of each PLN and tibia was calculated as one sample data. Data analysis was performed in a blinded fashion with regard to group allocations.

### Whole-mount immunofluorescence staining and confocal laser scanning of mice caudal skin

Due to the thickness of the skin of the leg, it is difficult to carry out whole mount immunofluorescence staining of this tissue. In order to observe whether LVs drain platelets, we established a caudal fracture model by transection of the mouse caudal vertebra with scissors. At the 1st and 7th day post-surgery, we removed the caudal skin from the fracture drainage site for whole mount staining. The tail skin was incubated with syrian hamster monoclonal anti-podoplanin antibody (Cat.No. ab11936, Abcam Inc., 1:1000) and rabbit monoclonal anti-CD41 antibody (Cat.No. ab134131, Abcam Inc., 1:1000) as primary antibodies, and Alexa Fluor 488 goat anti-hamster antibody (Cat. No. A-21110, Invitrogen, 1:200) and Alexa Fluor 488/555 goat anti-rat antibody (Cat.No. 4417S, Cell Signaling Technology, 1:1000) as secondary antibodies. After mounting, tissues were imaged with Olympus FV1000 confocal laser scanning microscope under a 40× objective. The exposure time, brightness and contrast of each image were applied equally across all images. The mean value of three magnification (×20) images of each LVs was calculated as one sample data. Data analysis was performed in a blinded fashion with regard to group allocations.

### Transmission electron microscopy

The isolated popliteal lymph nodes were fixed with 2.5% glutaraldehyde in 0.1M PBS, pH 7.4, for 24h. The specimens were post fixed in 1% buffered osmium tetroxide for 2h at 4°C, dehydrated through a graded series of ethanol, infiltrated/embedded into acetone/resin and polymerized at 60°C for 3 days. One-micron thick sections were then sliced. Then, 70nm thin sections were mounted onto 200 mesh carbon-coated nickel grids and stained with lead citrate for ultrastructural examination. The grids were examined and photographed using a FEI Tecnai G2 Spirit transmission electron microscope.

### Patients and sampling

Approval from the local institutional ethics committee of Long Hua hospital of Traditional Chinese Medicine was gained prior to this study (2018LCSY059). Briefly, this the prospective cohort study was designed to investigate the relationship between the lymphatic system and chronic musculoskeletal diseases, and breast cancer patients were included in the amendment, because we found that the upper limb joint inflammation of breast cancer patients after lymphadenectomy was closely related to lymphatic system dysfunction. Demonstrating LPT definitely occurring in patients with bleeding, cancer patients (n = 10) scheduled for LND were included randomized and incidentally by the general surgeon. Here, informed consent was given following the institutional guidelines (General Surgery Department, Long Hua hospital of Traditional Chinese Medicine). In addition to routine pathological examination of dissected LN specimens, immunofluorescence staining were applied to identify whether LPT exist. Patient information was listed in an independent supplementary table.

### Cannulation of the thoracic lymph ducts within the rat

In order to collect draining lymph from sham and fractured rats, we operated thoracic lymph duct cannulation before establishing rat tibial fracture or sham model^67^. Briefly, a midline abdominal incision was made approximately two thirds of the length of the abdomen posterior to the xiphoid cartilage. The thoracic lymph duct was separated away from the abdominal aorta, ligated with Two 4 – 0 silk and cannulated with a 15cm length of polyethylene tubing primed with heparinized saline. Lymph flow was seen as a white solution in the tubing. The polyethylene tubing was firmly fixed and passed through the peritoneum and skin by A 16G catheter and connected to the anticoagulant tube. After several peritoneal lavage, the peritoneum and skin were closed with sutures. Topical anesthetic cream was applied to the suture line on the rat. Replace anticoagulant tubes every 2 hours for draining lymph collection.

### Conditioned medium preparation

Rats were sacrificed respectively at day 1, 2 and 3 post-surgery. (a) For hematoma CM preparation, bone marrow (two bone marrows of tibial shafts from one rat) or hematoma tissue at fracture sites (two fractured tibial shafts from one rat) at day 1, 2 and 3 post-surgery were collected and homogenized with equal quality of DMEM/F-12 medium containing 10% FBS and 1% penicillin/streptomycin. The extracted medium were transferred to 1.5 mL Eppendorf tubes, centrifuged at 21,000 rpm for 15 minutes at 4 °C, and filtered with 0.22um sterile syringe filter. The filtered and collected medium was called hematoma CM and used to cell experiment and ELISA. (b) For 20% lymph CM preparation, collected thoracic duct lymph at day 1, and 2 post-sugery were diluted with 4 times volumes of DMEM/F-12 medium containing 10% FBS and 1% penicillin/streptomycin, centrifuged with 3000 rpm at 4 °C for 10min, and filtered with 0.22um sterile syringe filter. The filtered and collected medium was called 20% lymph CM and used to cell experiment and ELISA. (c) For 100% venous CM, collected rat femoral venous blood at day 1, 2, and 3 post-sugery were centrifuged with 3000 rpm at 4 °C for 10 min, and filtered with 0.22um sterile syringe filter. The filtered and collected medium was called 100% venous CM and used to cell experiment and ELISA.

### Osteoblasts preparation and identification

Osteoblasts isolated from newborn rat cranial bones of 1-day-old male SD Rats, as described previously^68^. The Cranial were washed with PBS containing 2% penicillin/streptomycin for three times, subsequently, periosteum and connective tissue between bone sutures were carefully removed under a stereo-microscope. Sections were cut into 1 cubic millimetre and digested six times by adding five times the volume of enzyme solution containing 0.1% collagenase (Gibco, Cat.No. 1148090) and 0.05% trypsin at 37°C for 20 minutes and repeating this procedure. Cells extracted from the last four to six steps were cultured in T-25 flasks (Corning) with DMEM/F-12 containing 10% foetal bovine serum (Gibco, Cat.No. 10099–141) at 5% CO2, 37°C. When the cell fusion reached 80%, the cells were removed from each flask and cultured in the same medium. The 3rd-5th generation were used for subsequent experiments.

### Bone marrow-derived BMSCs preparation and identification

BMSCs were isolated from the femurs and tibias of 4-week-old female SD rats from Vital River Laboratory Animal Technology (Beijing). As describe previously^69^, BMSCs were cultured in DMEM/F-12 with 10% FBS and 1% penicillin/streptomycin. After 48h, the adherent cells were transferred into fresh medium as 1st-passage cells. After 48 hours, the culture supernatant was removed, and the adherent growth of BMSCs was observed until for passaging. The 3–5th generation was used for cell identification and subsequent experiments. Continuously passage the cells as BMSCs fusion reached 80%. 3rd to 5th - passage were used for cell identification and phenotype.

For identification of the cells isolated from bone marrow, cell surface markers and multi-differentiation potential were analyzed. (a) Cell surface markers were analyzed by Flow cytometry. 3rd to 5th -passage cells were stained with MSC-related positive surface markers (CD44, CD90, CD29 and CD73) and negative surface markers (CD34 CD11b/c and CD45) using a Mesenchymal Stem Cell Surface Labeling Detection Kit (Cat No. RAXMX-09011, OriCell). Cells were subjected to flow cytometric analysis using a Accuri C6 Plus. Results were analyzed by Flowjo V10 data analysis software. (b) Alkaline phosphatase stainning and Alizarin Red staining for OB identification and Alcian blue staining for chondrocyte identification were used to confirm the multi-differentiation potential of BMSCs. 3rd -passage cells were induced and stained with related mesenchymal stem cells osteogenic differentiation kit (RAXMX-09021, OriCell) and mesenchymal stem cells chondrogenic differentiation Kit (RAXMX-90041, OriCell) according to the manufacturer’s instructions.

### Cell phenotypic experiments

Cell apoptosis assay by flow cytometry of Annexin V-APC/PI double-staining, cell growth by microscopic observation, and cell proliferation by cell counting and CCK8-Kit were assessed. (a) For flow cytometry of Annexin V-FITC/PI double-staining, OBs intervened by hematoma CM for 1 hour in (a) were prepared to be a single cell suspension (1×10^6^ cells/ml) with 1×buffer provided in the Annexin V Apoptosis Detection kit (eBioscience, 88–8007-74). 5 μl Annexin V-APC and 5 μl PI staining solution were added to 100 μl OBs suspension as one sample and all samples were incubated at room temperature for 10 min in the dark. A Attune CyPix Flow Cytometer(Invitrogen.) and the RL1:670-APC and YL1:585-PI channels were used to measure cell apoptosis, where the results were analyzed using the Flowjow V10 software. (b) For microscopic observation, a density of 3×10^4^ cells/well were seeded in 24-well plates, cultured for 12h and treated with or without CM for 1h at 37°C. The morphology and gross quantity of cell were photographed in 5 random high-power fields at ×100 magnification under microscope (Niko, Eclipse Ti2-U) for OBs and ×200 magnification under microscope for BMSCs. (c) For cell counting analysis, OBs/BMSCs at passages 3–5 were harvested and then seeded in 24-well plates at a density of 3×10^4^ cells/well. We cultured these cells with or without CM. After 24h of intervention, cells were harvested by removing the culture and adding 1 mL trypsin. After 1 min of digestion at room temperature, the collected cells were centrifuged at 1 000 rpm for 3 min with 5 mL of fresh medium and then resuspended in 1 mL of fresh medium. The final cells were counted by a Countstar^®^ Automated Cell Counter (Shanghai, China) with a 20 μL cell suspension. (d) For CCK-8 assay, a density of 3×10^3^ cells/well were seeded in 96-well plates, cultured for 12h and then treated with or without CM for 24h at 37°C. A cell counting kit 8 (Cat. No.#KJ798, Dojindo Laboratories) was used according to the manufacturer’s instructions.

### Identification of neutrophil and marcophage by flow cytometry

On 1–3 day post-surgery, hematoma tissues of fractured mice and bone marrow of sham mice are collected and prepared for singe cell suspension with a density of 1×106 cells/ml. The following antibodies are used to label myeloid cells:anti-F4/80-FITC (Cat.No.11–4801-82, Invitrogen), anti-CD11b-APC (Cat.No.17–0112-82, Invitrogen), anti-Ly6G-PE-Cyanine7 (Cat.No.A14748, Invitrogen), anti-Ly6C-PerCP/cy5.5 (Cat.No.A14801, Invitrogen) and Fixable Viability Dye eFluor^™^ 780 (Cat.No.65–0865-14, Invitrogen). The following antibodies are used to label M2-like macrophage:anti-F4/80-Percp-Cyanine5.5 (Cat.No.45–4801-80, Invitrogen), anti-CD11b-PE-PE-Cyanine5 (Cat.No.A26002, Invitrogen), anti-CD163-FITC (Cat.No.11–1631-82, Invitrogen). To avoid non-specific binding of dyes, the following isotype control antibodies are used: Rat IgG2a kappa Isotype Control (eBR2a)-FITC(11–4321-80,Invitrogen), Rat IgG2b kappa Isotype Control (eB149/10H5)-APC/PerCP-Cyanine5.5(17–4031-82/45–4031-80, Invitrogen/eBioscience).

### ELISA

Prepared hematoma CM, 100% venous CM and 20% lymph CM were used to measure the concentration of pro-inflammatory cytokines for IL-1β, IL-6 and TNF-α, growth factors for TGFβ and PDGF, HMW-DAMPs for HA, versican and HSP70, LMW-DAMPs for S100-A8 and HMGB1 using a ELISA Kit following the manufacturer’s instructions. ELISA plates was read using a microplate reader set at 450 nm wavelength. The following ELISA kits are used: IL-1β ELISA kit (Thermo, ER5RB), IL-6 ELISA kit (Thermo, 88–50625-88), TNF-α ELISA kit (Thermo, 88–7340-88), PDGF-BB ELISA kit (Proteintech, KE10034), TGF bata ELISA kit (Abisn, abs552208), Hyaluronan ELISA kit (Solarbio, SEKH-0509), Versican ELISA kit (Abbexa, abx518531), HSP70 ELISA kit (Abcam, ab133060), S100-A8 ELISA kit (CUDABIO, CSB-EL020641RA), HMGB1 ELISA Kit (Solarbio, SEKR-0074).

### 4D label-free quantitative proteome analysis

9 SD rats, having been successfully operated thoracic lymph duct cannulation, were randomly assigned to sham, VEH and CLO groups (n = 3/each group). Each group was carried out as described methods above. Draining lymph during 22th to 24th hour post-surgery was collected and detected by LC-MS/MS (Luming Bio, China). The MS/MS data was analyzed by MaxQuant search engine (v.1.6.15.0). FDR was adjusted to < 1%. The mass spectrometry proteomics data have been deposited to the ProteomeXchange Consortium (http://proteomecentral.proteomexchange.org) via the iProX partner repository with the dataset identifier PXD044917.

### mIHC and data analysis

Fresh frozen LN sections (7μm thick) was placed at room temperature for 10–20 minutes for rewarming. The prepared section was placed with Tris-EDTA buffer (pH 9.0), which was microwaved at high power for 5 minutes followed by an additional 10 minutes at low power for antigen retrieval. After cooled to room temperature (RT), slides were immersed with peroxidase-blocking solution for 5 minutes for blocking endogenous peroxidase activity and sealed with the 3% BSA for 30 minutes. The slides were subsequently incubated with the primary antibodies at 4°C overnight. Then, slides were washed thrice in 1X PBS buffer, and incubated with horseradish peroxidase (HRP)-conjugated secondary antibodies for 50 minutes at room temperature. A quick wash in 1X PBS buffer was followed by incubation with an appropriate fluorophore-conjugated tyramide signal amplification (TSA) for 10 min at RT. Again, the slides were exposed to microware treatment to strip the tissue-bound primary/secondary antibody complexes and ready for labelling of the next marker. The primary antibodies were incubated in following order: S100 (Abcam, ab288715, 1:500), Versican (Invitrogen, MA5–27638, 1:500), HSP70 (Abcam, ab181606, 1:500), HMGB1 (Abcam, ab79823, 1:500) and Podoplanin (Abcam, ab109059, 1:200). Corresponding HRP-conjugated secondary antibodies (WAS1201100) and fluorophore-conjugated TSA Kit (TSA570; TSA620; TSA650; TSA480; TSA690) were repeated until all markers were labelled. Finally, the slides were added with DAPI in the dark for 10 min at room temperature and mounted with anti-fade mounting medium. The PANNORAMIC MIDI II was used to take images of tissue samples.

### Statistical analysis

SPSS 20.0 software was used for statistical analyses. For measurement data, data are expressed as the means ± standard deviation (SD). All continuous numerical variables approximately accord with normal distribution test by kurtosis and skewness method. Comparisons between 2 different groups were analyzed using two-sided Student`s *t*-test. Paired *t*-test was used for the same group before and after comparison. Comparisons among 3 or more groups were performed by using one-way ANOVA and Bonferroni test if satisfied with homogeneity of variance, neither with one way ANOVA and Tamhane test. A repeated measurements study was analyzed by using repeated measurement analysis of variance. For count data, data are expressed as proportion, chi-square test or Fisher’s Exact Test was applied. *P < 0.05, **P < 0.01, and ***P < 0.001 means statistically significant difference.

## Data Availability

The data that support the findings of this study are available from the authors upon reasonable request.
